# Systemic Delivery of a Glucosylceramide Synthase Inhibitor Reduces CNS Substrates and Increases Lifespan in a Mouse Model of Type 2 Gaucher Disease

**DOI:** 10.1371/journal.pone.0043310

**Published:** 2012-08-17

**Authors:** Mario A. Cabrera-Salazar, Matthew DeRiso, Scott D. Bercury, Lingyun Li, John T. Lydon, William Weber, Nilesh Pande, Mandy A. Cromwell, Diane Copeland, John Leonard, Seng H. Cheng, Ronald K. Scheule

**Affiliations:** Genzyme Corporation, Framingham, Massachusetts, United States of America; Weizmann Institute of Science, Israel

## Abstract

Neuropathic Gaucher disease (nGD), also known as type 2 or type 3 Gaucher disease, is caused by a deficiency of the enzyme glucocerebrosidase (GC). This deficiency impairs the degradation of glucosylceramide (GluCer) and glucosylsphingosine (GluSph), leading to their accumulation in the brains of patients and mouse models of the disease. These accumulated substrates have been thought to cause the severe neuropathology and early death observed in patients with nGD and mouse models. Substrate accumulation is evident at birth in both nGD mouse models and humans affected with the most severe type of the disease. Current treatment of non-nGD relies on the intravenous delivery of recombinant human glucocerebrosidase to replace the missing enzyme or the administration of glucosylceramide synthase inhibitors to attenuate GluCer production. However, the currently approved drugs that use these mechanisms do not cross the blood brain barrier, and thus are not expected to provide a benefit for the neurological complications in nGD patients. Here we report the successful reduction of substrate accumulation and CNS pathology together with a significant increase in lifespan after systemic administration of a novel glucosylceramide synthase inhibitor to a mouse model of nGD. To our knowledge this is the first compound shown to cross the blood brain barrier and reduce substrates in this animal model while significantly enhancing its lifespan. These results reinforce the concept that systemically administered glucosylceramide synthase inhibitors could hold enhanced therapeutic promise for patients afflicted with neuropathic lysosomal storage diseases.

## Introduction

Gaucher Disease results from a deficiency of the lysosomal enzyme glucocerebrosidase (GC). In the most common phenotype of Gaucher disease (type 1), pathology is limited to the reticuloendothelial and skeletal systems [1] and there are no neuropathic symptoms. In neuropathic Gaucher disease (nGD), subdivided into type 2 and type 3 Gaucher disease, the deficiency of glucocerebrosidase (GC) causes glucosylceramide (GluCer) and glucosylsphingosine (GluSph) to accumulate in the brain, leading to neurologic impairment. Type 2 Gaucher disease is characterized by early onset, rapid progression, extensive pathology in the viscera and central nervous system, and death usually by 2 years of age. Type 3 Gaucher disease, also known as subacute nGD, is an intermediate phenotype with varying age of onset and different degrees of severity and rates of progression [2]. A recent development has produced the K14 lnl/lnl mouse model of type 2 Gaucher disease (hereinafter referred to as K14); this mouse model closely recapitulates the human disease, showing ataxia, seizures, spasticity and a median lifespan of only 14 days. [3].

As in patients with nGD, several mouse models of the disease have increased levels of GluCer and GluSph in the brain due to the deficiency in GC activity [4], [5]. A homozygous GC knockout mouse presents with an ∼100-fold elevation of GluSph in the brain as early as day 13 of gestation, and these levels increase until early neonatal death from disruption of the epithelial barrier of the skin [3], [6]. An increase in GluSph has also been observed in human fetuses with type 2 Gaucher disease, thus confirming the similarity of the pathologic processes in mice and humans [7]. Restricting GC expression to the skin with a keratin-14 promoter helped overcome the early mortality observed in previous mouse models of the disease. The resulting “K14” mice display a neuropathic phenotype that shares many pathologic features with type 2 Gaucher disease, such as neurodegeneration, astrogliosis, microglial proliferation, and increased levels of GluCer and GluSph in specific brain regions [3].

Clinical management of patients affected by nGD poses a challenge for treating physicians both because of the severity of type 2 disease and the inability of the current therapies to cross the blood brain barrier (BBB). In type 3 Gaucher disease, treatment using high doses of intravenous recombinant human glucocerebrosidase (rhGC) has been evaluated [8]. Even though this treatment is useful for reducing visceral disease, it has not provided convincing evidence of its ability to reduce the rate of progression of the neurological symptoms [9], [10]. Recent studies have explored the possibility of directly administering lysosomal enzymes to the brain to circumvent the BBB, and have shown efficient biodistribution, clearance of substrate, amelioration of pathology and behavioral improvements in mouse models of Neuronal Ceroid Lipofuscinosis, Niemann-Pick disease and Gaucher disease [11], [12]. Indeed, we have shown recently that brain pathology and survival in the K14 Gaucher mouse model could be enhanced by intracerebroventricular injection of GC in neonatal animals [13].

Miglustat, a marketed, non specific glucosylceramide synthase inhibitor (GSI) which crosses the BBB does not appear to address the neuropathic symptoms of nGD. To this point, we have shown in a mouse model of Sandhoff disease [14] that *N*B-DNJ (active ingredient in miglustat) counterintuitively increases brain GluCer levels, while GZ 112638 a specific GSI in clinical trials, which does not cross the BBB, has no effect as expected. To identify a glucocerebroside synthase (GCS) inhibitor with the ability to cross the BBB, we screened a series of novel synthetic compounds, resulting in the nomination of GZ 161 as a potential therapeutic. An evaluation of GZ 161 in the K14 mouse model of type 2 Gaucher disease demonstrated that it could indeed reduce brain GluCer and GluSph. It also reduced brain neuropathology and extended the lifespan of this model. Although this substrate reduction approach may have promise for type 3 Gaucher disease, in the K14 model inhibiting GCS in this way did not appear to be as efficacious as supplying GC directly to the murine brain. Thus a combined approach using both enzyme replacement and small molecule substrate reduction may, at least in principle, represent a more effective approach for neuropathic Gaucher disease.

## Results

**Figure 1:**
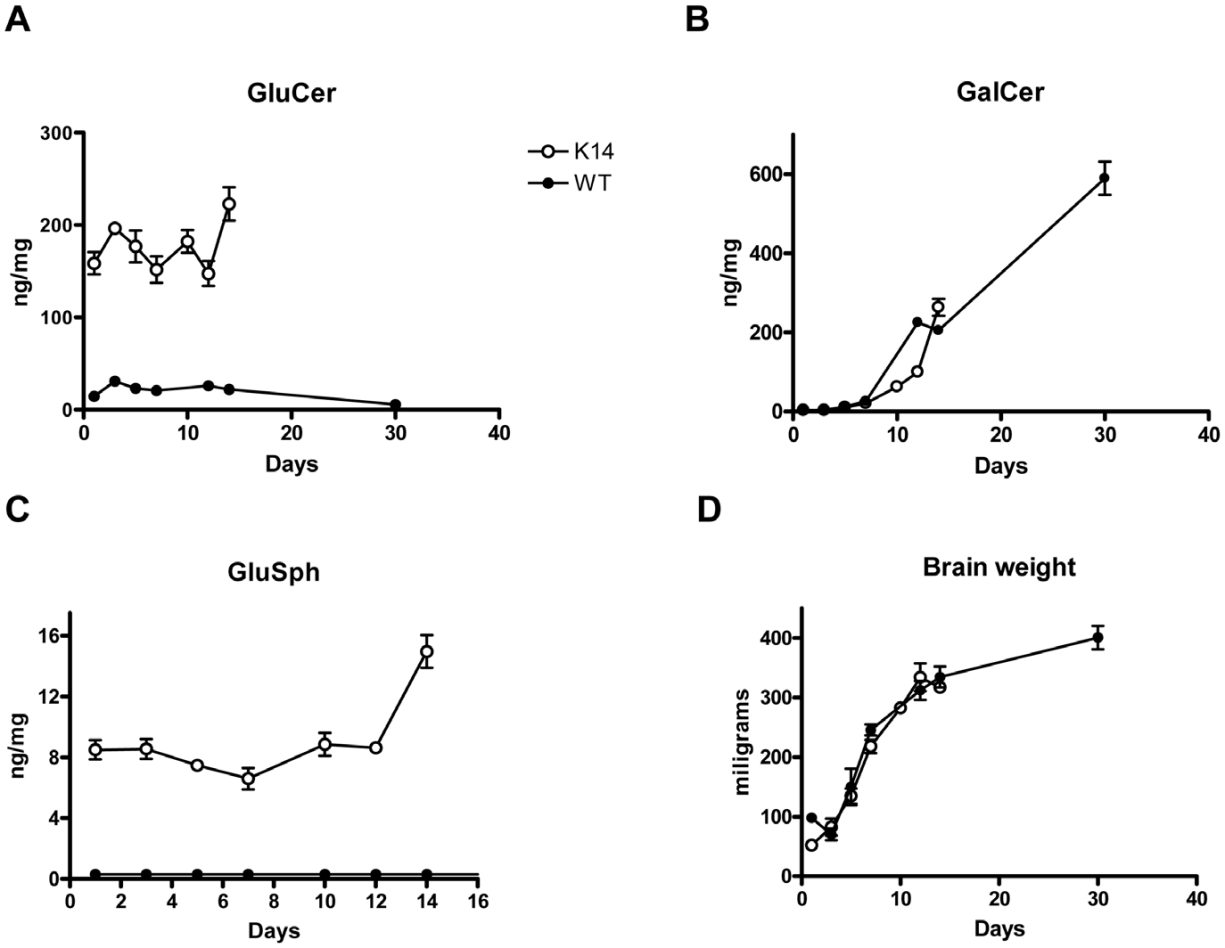
GluCer and GluSph are significantly elevated in the brains of neonatal K14 mice. Mass spectrometry analysis of glucosyl- and galactosylceramides shows that (A) GluCer was elevated 10-fold in K14 mice compared to WT mice through the first 2 weeks of life, (B) GalCer levels were similar over time for both K14 and WT mice, (C) GluSph levels were ≥10-fold higher in K14 mice than age-matched WT mice over the first 2 weeks of life; GluSph levels in WT animals were below the level of detection (<0.3 ng/mg). (D) There were no significant differences in brain weights between K14 and WT mice over the first 2 weeks of life. Data points represent mean values and error bars SEM for N = 4.

Before evaluating drug effects on brain lipids, we compared the time dependent changes in GluCer, GalCer and GluSph levels in the K14 mouse brain to those of a wild type (WT) mouse control. Figures 1A and 1B show that in WT mouse brain, the predominant GL-1 isomer in the first few days of life was GluCer; by postnatal day 14 (P14) the predominant isomer was GalCer. These results are consistent with those of a study in rat brain, which found that GluCer is synthesized at a higher rate during the first week of life and is followed by an increased synthesis of GalCer starting at P8 [15]. Figure 1A also shows that in K14 mice GluCer was elevated 10-fold relative to WT mice and that this increase was sustained through the first 2 weeks of life until the mice died around P14.

In agreement with previous mouse models of neuropathic Gaucher disease [4], Figure 1C shows that at birth the lysoglycosphingolipid GluSph was elevated >20-fold in the brains of the K14 mouse model relative to WT mice. This increase was sustained through the first 2 weeks of life and was even higher in animals sacrificed at end stage (Fig. 1C). In WT littermates of the K14 mice, GluSph levels were below the threshold of detection (0.3 ng/mg of tissue). Figure 1D shows that these elevated glycosphingolipids and lysoglycosphingolipids in the K14 mouse did not appear to have an impact on brain weight (relative to that of WT mice). Given the known toxicity of GluSph, therapeutic strategies geared towards reducing the accumulation of these substrates in the K14 mouse brain might be expected to have an impact on the pathologic features of the disease and the lifespan of the animals.

### Intraperitoneal Administration of GZ 161 Reduces GluCer and GluSph Levels in the Brains of K14 mice

**Figure 2:**
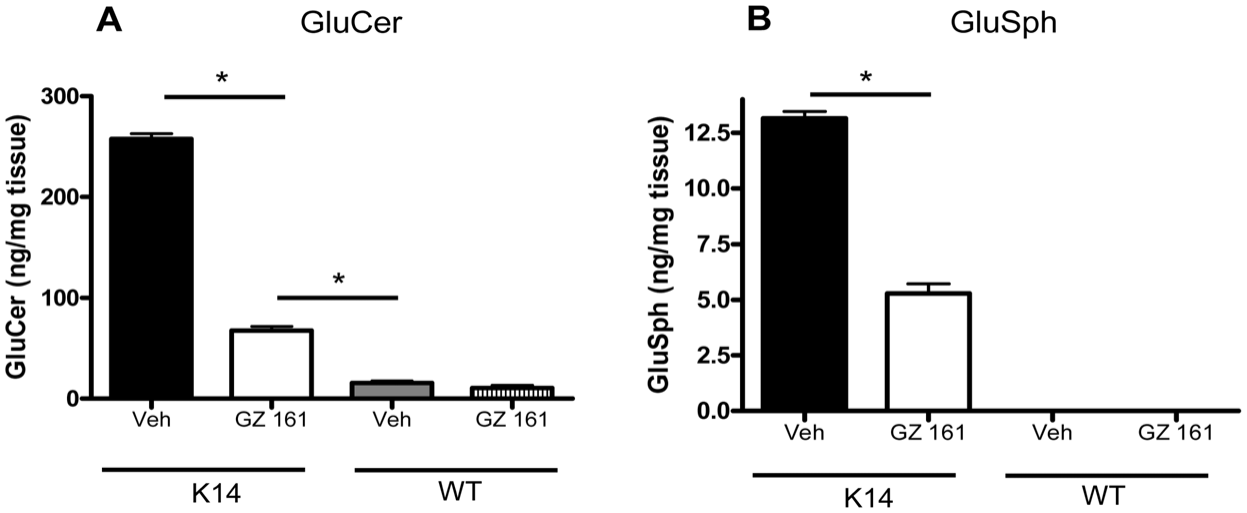
Systemic administration of GZ 161 reduces GluCer and GluSph levels in the K14 mouse brain. K14 and WT mice were treated daily (IP) beginning at P4 with vehicle or 5 mg/kg GZ 161, and brains analyzed for GluCer and GluSph at P10. GZ 161-treated animals were asymptomatic at this time. Treatment with GZ 161 reduced K14 (A) GluCer levels by ∼70% and (B) GluSph levels by ∼60%. Post-treatment levels of both glycosphingolipids remained significantly elevated compared to their WT littermates. and genotypes were confirmed by post-mortem DNA analysis. *p<0.05. N = 4/group.

Figure 2 shows that compared to vehicle-treated K14 mice at the humane endpoint (14–15 days of age), daily intraperitoneal (IP) administration of GZ 161 reduced brain levels of both GluCer and GluSph by >60%. K14 mice treated with GZ 161 were asymptomatic at this time point. Even though GZ 161 administration significantly reduced the levels of these glycosphingolipids, Figure 2 shows that they nonetheless remained elevated several-fold over those of age-matched WT mice; GluSph was not detected in samples analyzed from WT or heterozygote littermates. The reduction of brain glycosphingolipids as a consequence of systemic drug administration strongly suggests that GZ 161 is both capable of crossing the blood brain barrier and inhibiting its target enzyme, GCS.

### Intraperitoneal Administration of GZ 161 Reduces Microglial/macrophage Staining throughout the Brain of K14 mice

Cells of the myeloid lineage can be detected in the murine brain using antibodies to antigens such as F4/80 and CD68. F4/80 is a transmembrane glycoprotein found on ramified (quiescent) microglia [16] and macrophages, while CD68 is a lysosomal protein expressed at relatively high levels in macrophages and activated (reactive) microglia and at lower levels in ramified microglia [17], [18], [19]. Increased F4/80 and CD68 staining in the brain may occur through recruitment of monocytes or microglial proliferation [20] and is a normal response to injury and inflammation.

**Figure 3:**
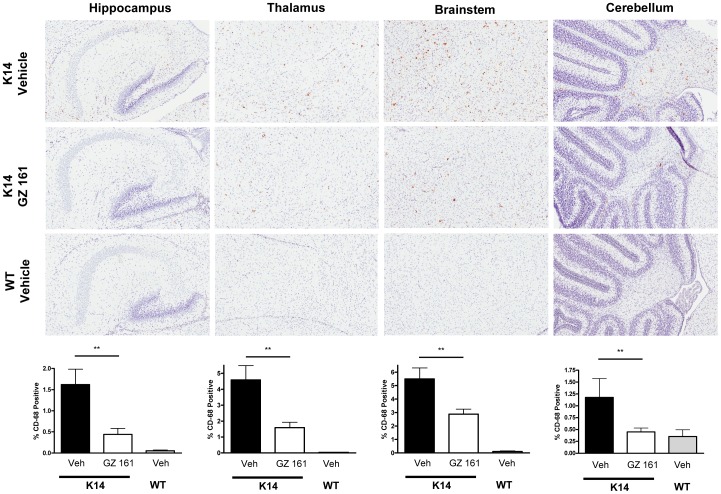
Systemic administration of GZ 161 reduces CD68 staining throughout the brain of K14 mice. (Upper panels) Representative immunohistochemical CD 68 staining at P10 in the hippocampus, thalamus, brainstem and cerebellum of K14 mice treated daily (IP) beginning at P4 with vehicle or GZ 161 and WT mice treated with vehicle. (Lower panels) Quantitation of staining in the groups shown above, showing that systemic treatment with GZ 161 results in significant reductions the CD68+ cells in all brain regions. Similar reductions were observed in other structures such as the olfactory bulb and frontal cortex (data not shown). **p<0.01. N = 4/group.

**Figure 4:**
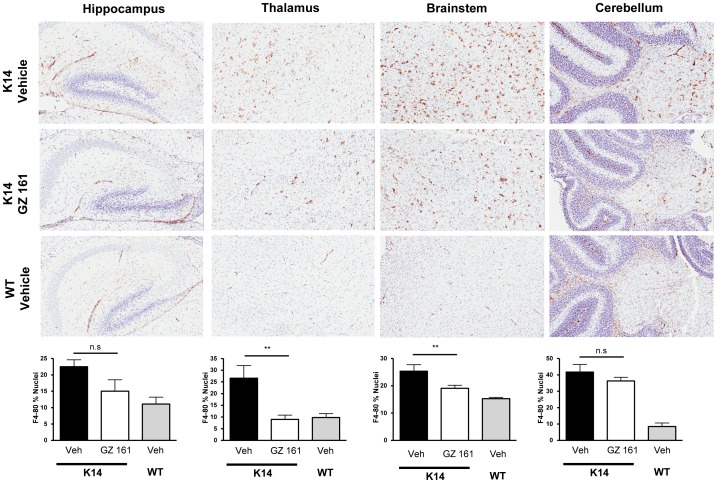
Systemic administration of GZ 161 reduces F4/80 staining in some brain regions of K14 mice. (Upper panels) Representative immunohistochemical F4/80 staining at P10 in the hippocampus, thalamus, brainstem and cerebellum of K14 mice treated daily (IP) beginning at P4 with vehicle or GZ 161, and WT mice treated with vehicle. (Lower panels) Quantitation of staining in the groups shown above, showing that systemic treatment with GZ 161 results in significant reductions the F4/80+ cells in the thalamus and brainstem. Similar reductions were observed in other structures such as the olfactory bulb and frontal cortex; statistical differences were observed in both structures (data not shown). *p<0.05. N = 4/group.

Figure 3 shows qualitatively and quantitatively that compared to wild type mice at 10 days of age (P10), the K14 mouse brain has increased numbers of CD68+ cells in multiple locations (hippocampus, thalamus, brainstem, cerebellum). The greatest concentration of CD68+ cells was seen in the thalamus and brainstem, two sites that also show pathology in type 2 Gaucher patients [21], [22], [23]. Figure 3 also shows that systemic administration of GZ 161 reduces the numbers of CD68+ cells in all of these locations; treatment also reduced CD68+ cells in the olfactory bulb and frontal cortex (data not shown). Consistent with the CD68 histopathology, Figure 4 shows increased F4/80 staining relative to WT animals in vehicle treated K14 mice at P10. Daily IP injections of GZ 161 reduced the numbers of F4/80+ cells in the thalamus and brainstem, but had marginal effects in other brain regions. Taken together with the CD68 data, these results suggest that systemic treatment of the K14 mouse with GZ 161 results in decreased numbers of macrophages/microglia in multiple brain regions. Whether these decreases are stable relative to vehicle treated K14 mice or simply indicate a delay in the accumulation of these cells in the brain is not clear.

### Intraperitoneal Administration of GZ 161 Reduces Gliosis in Several Brain Regions of K14 mice

**Figure 5:**
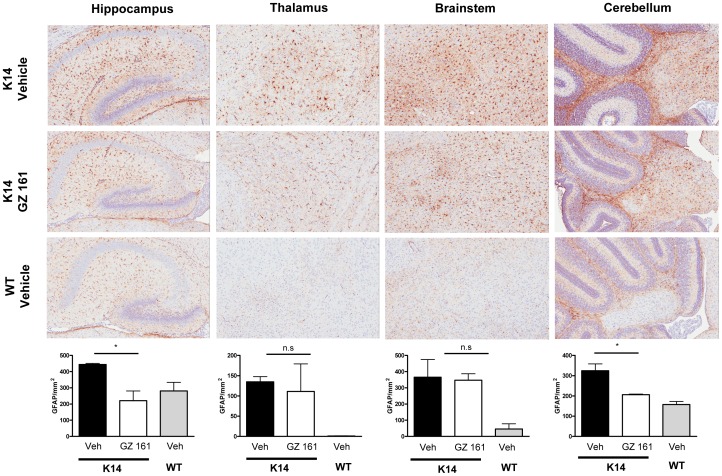
Systemic administration of GZ 161 decreases gliosis in K14 mice. (Upper panels) Representative immunohistochemical GFAP staining at P10 in the hippocampus, thalamus, brainstem and cerebellum of K14 mice treated daily (IP) beginning at P4 with vehicle or GZ 161, and WT mice treated with vehicle. (Lower panels) Quantitation of staining in the groups shown above, showing that systemic treatment with GZ 161 results in significant reductions the GFAP+ cells in the hippocampus and cerebellum; statistical differences were observed in both structures (data not shown).

Astrocytes can undergo hypertrophy or proliferate in response to inflammation and neuronal damage or death, a process known as astrogliosis. Glial fibrillary acidic protein (GFAP) is an intermediate filament protein that is heavily expressed in activated (reactive) astrocytes, and can therefore be used to monitor astrogliosis. Figure 5 shows that at P10 GFAP staining was increased compared to WT levels in several brain regions (hippocampus, thalamus, brainstem, cerebellum) of the K14 mouse, indicating the presence of reactive astrocytes. Figure 5 also shows that systemic treatment of K14 mice with GZ 161 led to decreased GFAP staining in the hippocampus and cerebellum at P10; staining was also decreased in the olfactory bulb and frontal cortex (data not shown). Thus, these GFAP results are consistent with the above macrophage/microglial data demonstrating that the K14 mouse likely has an ongoing inflammatory process that can be attenuated to some degree by systemic administration of GZ 161.

### Intraperitoneal Administration of GZ 161 Increases Survival of K14 mice

**Figure 6:**
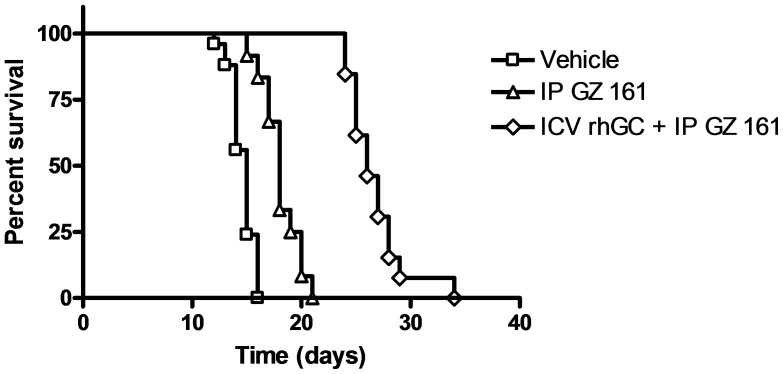
Systemic administration of GZ 161 increases the median lifespan of K14 mice. K14 mice were injected (IP) daily beginning at P4 with vehicle or GZ 161 or given a combined treatment of three intracerebroventricular (ICV) injections of rhGC at P1,2,3 together with daily (IP) injections of GZ 161 beginning at P4. Vehicle treated mice had a 15 day median lifespan (N = 25); GZ 161 treated mice had an 18 day median lifespan (N = 12; p<0.0001 compared to vehicle-treated); mice administered GZ 161 and rhGC had a 26 day median lifespan (N = 13).

Given the positive effects of GZ 161 treatment on brain glycosphingolipids and histopathology, we asked whether these effects translated into increased survival of the K14 mouse. Figure 6 demonstrates that vehicle treated K14 mice have a median lifespan of 15 days, consistent with our previous findings in this mouse model [13]. Systemic (IP) treatment of K14 mice with GZ 161 resulted in an extension of median lifespan to 18 days (p<0.0001), consistent with a benefit of the molecular and cellular effects of the drug in the brain shown above.

We have previously shown in the K14 mouse that neonatal (P1–P3) intracerebroventricular injections of GC could extend median survival even further, viz., to 23 days [14]. Because GC and GZ 161 both have the potential to decrease levels of the same glycosphingolipid, namely GluCer (GC by degrading GluCer; GZ 161 by inhibiting its synthesis) we also asked whether the combination of GZ 161 and intracerebroventricular (ICV) administration of GC would provide survival benefit superior to that resulting from either individual agent. Figure 6 demonstrates that the combination of ICV GC (at P1,2,3) and daily IP GZ 161 led to a median survival of 26 days, significantly greater than GZ 161 alone or ICV GC (p = 0.0007). Thus, the survival benefits of systemically administered GZ 161 appear to be additive to those of ICV rhGC.

### Prenatal Administration of GZ 161 Fails to Increase Survival of K14 mice

Because the GluSph levels in the K14 mouse brain were found to be elevated at least 10-fold over normal at P1, and it has been documented that GluSph is elevated in the brains of mice and humans affected by nGD even prenatally [7], we also asked whether a survival advantage could be gained by treating K14 mice with GZ 161 *in utero*. Figure S1 shows that treating WT mouse dams with GZ 161 led to an ∼5-fold decrease in GluCer levels in the newborn mouse brain (P0), suggesting that GZ 161 could cross the blood/placental barrier. However, giving GZ 161 to pregnant heterozygote females and then treating the resulting pups IP with GZ 161 failed to extend survival beyond that of mice given systemic GZ 161 postnatally alone (18 days) (Figure 6 and Figure S2). These data are thus consistent with the results described in Figure 6, and imply that although GZ 161 can effect reductions in glycosphingolipids and neuropathology, the current treatment regime is insufficient to rescue the CNS. These results are consistent with our previous results in this model using intracerebroventricular injections of recombinant human glucocerebrosidase [13], and together suggest that more robust and continuous depletion of glycosphingolipids such as GluCer will be necessary to improve survival further.

## Discussion

The intravenous use of commercial forms of recombinant human glucocerebrosidase is regarded as the gold standard for the treatment of Gaucher disease, and provides significant improvement in the hematological, skeletal, visceral and quality of life outcomes of patients [24]; substrate inhibition approaches are also included in the therapeutic modalities available for Gaucher disease and have lately shown promising results [25]. Current experience using high doses of rhGC for treating neuropathic Gaucher disease has yielded mixed results, and has not confirmed the avoidance or stabilization of the neuropathic features of the disease in type 3 Gaucher disease patients [9]. As one potential therapeutic approach, we have shown recently that direct ICV administration of rhGC to the lateral ventricles of a mouse model of type 2 nGD, namely the K14 mouse, facilitates the clearance of glycosphingolipid substrates and improves median lifespan [13]. The identification of GZ 161 as a GCS inhibitor that can cross the BBB prompted us to evaluate this molecule in this same mouse model as a potential alternative or adjunct to other therapeutic approaches, such as ICV delivery of GC.

Here we show both qualitatively and quantitatively that systemic (IP) administration of GZ 161 to neonatal K14 mice significantly reduces substrate load, ameliorates the pathological features of the disease and increases median lifespan. When combined with ICV-delivered rhGC, systemic administration of GZ 161 resulted in additive increases in lifespan, implying that such a combination might be more efficacious than either monotherapy alone in nGD patients. Given the implications of these studies that GZ 161 can apparently cross the BBB and inhibit its target enzyme, glucosylceramide synthase, it is reasonable to assume that this molecule could also be used to treat other LSDs resulting from a buildup of substrates downstream from GluCer.

It is important to note that in the current studies, GZ 161 was administered to K14 mice in a time frame in which GluCer and GluSph were being produced in the developing mouse brain at relatively high rates compared to WT mice (Figure 1) [15]. Daily IP treatment with GZ 161 successfully reduced, but did not normalize GluCer and GluSph levels in the K14 brain (Figure 2). There are several lines of evidence suggesting that GluSph and other lysosphingolipids such as galactosylsphingosine may contribute to CNS pathology by initiating the production of inflammatory mediators [26], [27]. The ability of GZ 161 to decrease GluSph levels and concurrently result in decreased macrophage/microglial and astrocyte staining (Figures 3, 4, and 5) is consistent with this hypothesis. Because GluSph also has known neurotoxic properties [28], [29], [30], [31], the inability of GZ 161 treatment to normalize GluSph levels is consistent with GluSph as a potential contributor to the early death seen in this model.

Taken together, the preclinical results in the K14 mouse model shown here suggest that systemic administration of GZ 161 may mitigate disease progression and neurologic symptoms in type 2 and type 3 Gaucher disease patients. However, it is difficult to predict the potential benefits of such a therapeutic approach in symptomatic type 2 patients since it is known that their brains contain very high levels of GluSph that date back to prenatal life [2]. Type 3 Gaucher disease may be more amenable to treatment since the brain levels of GluSph are lower [32], the progression of the disease is slower despite being part of a phenotypic continuum [2], and in some cases the patients can be identified by mutational analysis before the onset of the neuropathic phenotype [33]. Based on the current results, it would appear that an early, aggressive approach will be needed to treat these patients. To this end, small molecule inhibitors of glucosylceramide synthase may represent one arm of a comprehensive approach.

## Materials and Methods

### Animals

Ethics Statement: Procedures involving mice were reviewed and approved by Genzyme Corporation’s Institutional Animal Care and Use Committee (Protocol 07-1115-2-BC) following guidelines established by the Association for Assessment of Accreditation of Laboratory Animal Care. The review board specifically approved all the studies (identification numbers 08-2932 and 09-4175) reported in this manuscript.

K14 lnl/lnl (abbreviated as K14) mice were obtained from Lund University [3] and bred under a protocol approved by the Institutional Animal Care and Use Committee. Treatments were administered as described in the manuscript and the animals were humanely sacrificed at pre-determined time points or upon reaching a humane endpoint defined as the inability to right 10 seconds after placed in lateral recumbency or loss of body weight of more than 15%.

Pups obtained from heterozygote mating were tail clipped and genotyped within one day of birth (by P1). The DNA was extracted using a lysis buffer of 5 mM EDTA, 0.2%SDS, 200 mM NaCl, 100 mM Tris pH 8.0 supplemented with 0.25 mg/ml Proteinase K (Invitrogen, Carlsbad, California), precipitated with 100% isopropanol and redissolved in 1X Tris EDTA buffer. The DNA was then used for polymerase chain reaction (PCR) to determine the presence of the GC gene under the K14 keratin promoter (CRE) [3]. To determine the Neomycin resistance site disruption of the murine glucocerebrosidase gene (NEO) we used a three primer approach: GC WT Fwd 5′-TGTTCCCCAACACAATGCTCTTT-3′; Rev 5′-TCTGTGACTCTGATGCCACCTTG-3′ and Neo Rev 5′-AAGACAGAATAAAACGCACGG GTG-3′ as previously described [13].

### GZ 161 Dosing

Based on preliminary studies of GZ 161, it was found that adult wild type mice tolerated doses of up to 60 mg/kg/day and as low as 3 mg/kg/day were effective in reducing GluCer concentrations in the brain and viscera of WT mice (data not shown). However, in newborn mice it was found that the highest tolerated dose was 5 mg/kg/day. This dose was therefore used for our studies. Newborn mice received daily 5 mg/kg intraperitoneal injections of GZ 161 once a day in a volume of 10 µl/g of body weight starting at postnatal day 4. A subset of mice was continuously treated with GZ 161 and enrolled into a survival study where they were sacrificed when reaching a humane endpoint. To determine the effects of GZ 161 in substrate accumulation and the histopathological features of the mice, K14 mice and wild type littermates were sacrificed at postnatal day 10, which is the time at which symptoms are expected in untreated K14 mice. Mice received a 150 mg/kg dose of pentobarbital (Euthasol, Virbac Inc, Forth Worth, TX) and were transcardially perfused with cold 0.9% NaCl solution. Brains were dissected and divided; one hemisphere was used for GSL analysis and the other was fixed in 4% paraformaldehyde for 96 hours and processed for histology.

To determine if further benefits could be achieved by prenatal exposure to GZ 161, a subset of pregnant K14 females received GZ 161 in food using a formulation calculated to provide 20 mg/kg/day during the final 5–7 days of gestation. Females receiving GZ 161 were switched to standard diet after delivery and the pups received daily IP injections of GZ 161 at a dose of 5 mg/kg (10 µl/g of body weight) starting at P1. A set of WT pups born to females receiving the drug or standard formula was sacrificed immediately after birth to determine whether *in utero* exposure to GZ 161 could reduce brain GSL levels.

### Glycosphingolipid Quantitation

Quantitative sphingolipid analysis was performed by liquid chromatography and tandem mass spectrometry (LC/MS/MS) as previously described [34]. Briefly, 10 µl of brain tissue homogenate (tissue weight/water:100 mg/ml) was extracted with 1.00 ml of an organic solvent mixture (97% acetonitrile, 2% methanol, and 1% acetic acid, v/v) and vortexed vigorously for 10 min. Extracted sphingolipids (GluCer and GluSph) were directly separated by hydrophilic liquid chromatography (Atlantis HILIC column, Waters Corp.) and analyzed by triple quadrupole tandem mass spectrometry (API 4000, Applied Biosystems/MDS SCIEX) and compared with sphingolipid standards (Matreya, LLC; Pleasant Gap, PA).

### Reformulation of Recombinant Human Glucocerebrosidase

Recombinant human glucocerebrosidase (rhGC) was formulated for CNS administration as previously described [13]. Briefly, rhGC was bound using a cation-exchange (CM Sepharose) and human serum albumin (HSA) was added to the eluate as a stabilizer. The formulation for ICV administration was 2 mg/ml rhGC in a 10 mM sodium phosphate buffer at pH 7.2 containing 135 mM sodium chloride, 5 mg/ml HSA and 0.01% polysorbate 80.

### Intracerebroventricular Injections

Animals identified as K14 were cryoanesthesized and received 2 µl bilateral intracerebroventricular (ICV) injections of either rhGC at 2 mg/ml or vehicle as previously described [13]. The injected pups were monitored for recovery and returned to the mother following the procedure.

### Histopathology

After genotype confirmation, animals were humanely sacrificed at 10 days of age, At this age K14 mice are asymptomatic. Mice received an intraperitoneal injection of 150 mg/kg sodium pentobarbital (Euthasol, Virbac Inc, Forth Worth, TX) and were perfused by an intracardial infusion of chilled 0.9% sodium chloride. Brains were removed and post fixed in 4% paraformaldehyde for 72 hours. Tissue was transferred to PBS and paraffin embedded. Sagital sections 5 µm thick were cut and stained as described below. Gliosis and the presence of cells of the macrophage lineage were evaluated by means of glial fibrillary acidic protein staining and expression of CD68 and F4/80 pan-macrophage markers using the Leica Bond Max Immunostainer system (Leica Microsystems, Wetzlar, Germany).

#### GFAP staining

Paraffin sections were placed on mounting slides and processed using the Bond Polymer Refine IHC system (Leica Microsystems, Wetlzar, Germany) blocked for 10 minutes in serum-free protein block (Dako systems, Glostrup, Denmark), incubated for 30 minutes in a 1∶1500 dilution of primary anti-GFAP antibody in Dako antibody diluent (Dako, Glostrup, Denmark), and stained using the Bond Polymer Refine detection kit (Leica Microsystems, Wetzlar, Germany).

#### F4/80 staining

Paraffin sections were placed on mounting slides and processed using the Bond Polymer Refine IHC system (Leica Microsystems, Wetlzar, Germany), incubated for 30 minutes in a 1∶2500 dilution of rat anti- mouse F4/80 antibody (eBioscience, San Diego, CA) or Rat IgG2a (eBioscience, San Diego, CA) as an isotype control. Slides were then incubated with a 1∶250 dilution of rabbit anti-rat secondary antibody (Vector laboratories, Burlingame, CA) and stained using the Bond Polymer Refine detection kit (Leica Microsystems, Wetzlar, Germany).

#### CD 68 staining

Paraffin sections were placed on mounting slides and processed using the Bond Polymer Refine IHC system (Leica Microsystems, Wetlzar, Germany), incubated for 30 minutes in a 1∶2500 dilution of rat anti- mouse CD68 clone FA-11 antibody (AbD Serotec, Oxford, UK) or Rat IgG2a isotype control (AbD Serotec, Oxford, UK). Slides were then incubated with a 1∶250 dilution of rabbit anti-rat secondary antibody (Vector laboratories, Burlingame, CA) and stained using the Bond Polymer Refine detection kit (Leica Microsystems, Wetzlar, Germany).

For each staining technique exposure-matched digital images were obtained from similar brain regions of each experimental group using the Aperio ScanScope XT system (Aperio Technologies, Vista, CA). Stained slides were digitalized in high resolution and six areas of interest were highlighted in each slide and analyzed independently by histomorphometry. Positively stained area and nuclei were determined and quantitative data were analyzed by a one-way analysis of variance followed by Tukey’s multiple comparison test using the Graph Pad Prism V 4.0 (GraphPad Software, San Diego, CA). Differences between group means with p<0.05 were considered significant.

### Survival

K14 mice received daily intraperitoneal injections of GZ 161 at a dose of 5 mg/kg of body weight as described above. A separate cohort of animals also received ICV injections of GC at postnatal days 1, 2 and 3 followed by daily IP injections of GZ 161. Animals that reached weaning age received GZ 161 in a special chow designed to provide a dose of 60 mg/kg/day. All animals were monitored daily for the development of neurological complications. Mice were sacrificed when they reached a humane endpoint (inability to right within 10 seconds after being placed in lateral recumbence) by an injection of 150 mg/kg sodium pentobarbital (Euthasol, Virbac Inc, Forth Worth, TX). This time point was recorded as end of life and analyzed using Kaplan-Meier plots.

### Statistical Analysis

Values shown correspond to means and error bars represent standard error of the mean. Comparisons between groups were analyzed by a one-way analysis of variance followed by Tukey’s multiple comparison test. Comparison of substrate reduction *in utero* was analyzed by the unpaired t test with Welch’s correction. Kaplan-Meier survival curves were analyzed using the log-rank test equivalent to the Mantel-Haenszel test. All statistical analyses were performed using GraphPad Prism v4.0 (GraphPad Software, San Diego, CA). Differences between group means with p<0.05 were considered significant.

## Supporting Information

Figure S1 **GZ 161 appears to cross the blood/placental barrier.** Systemic administration (20 mg/kg/day in food) of GZ 161 to pregnant WT mice reduces the GluCer load in whole brain homogenates of mice at birth (P0). N = 7; p<0.0001).
(TIF)

Figure S2 **Treating K14 mice with GZ 161** ***in utero*** **has a minimal effect on survival.** K14 mice treated daily (IP) beginning at P4 with vehicle had a median lifespan of 14 days (N = 13). Systemic administration (20 mg/kg/day in food) of GZ 161 to pregnant heterozygote females and then daily systemic (IP) administration of GZ 161 (5 mg/kg) to the pups beginning at P0 extended lifespan to 19 days (N = 13), a result similar to treating pups daily systemically (IP) with GZ 161 at 5 mg/kg beginning at P4 (N = 12).
(TIF)
