# Supplementary figures and images for: Systemic Delivery of a Glucosylceramide Synthase Inhibitor Reduces CNS Substrates and Increases Lifespan in a Mouse Model of Type 2 Gaucher Disease
Source: PLoS One. 2012 Aug 17;7(8):e43310. doi: 10.1371/journal.pone.0043310 (PMC3422338; doi:10.1371/journal.pone.0043310)

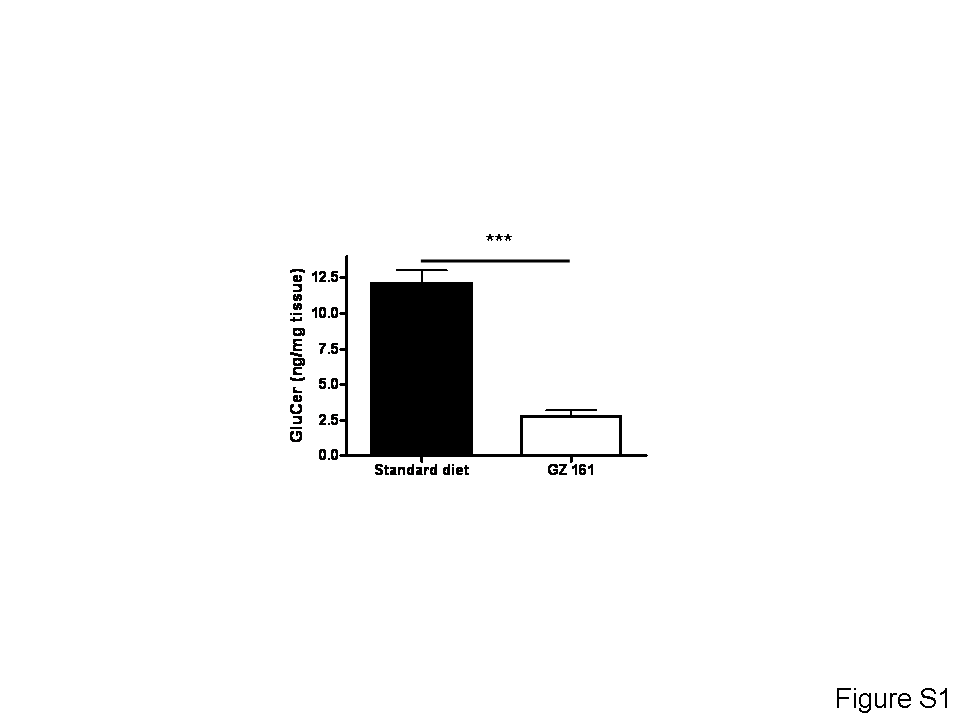

Supplement: Figure S1 — GZ 161 appears to cross the blood/placental barrier. Systemic administration (20 mg/kg/day in food) of GZ 161 to pregnant WT mice reduces the GluCer load in whole brain homogenates of mice at birth (P0). N = 7; p<0.0001). (TIF) [file pone.0043310.s001.tif]

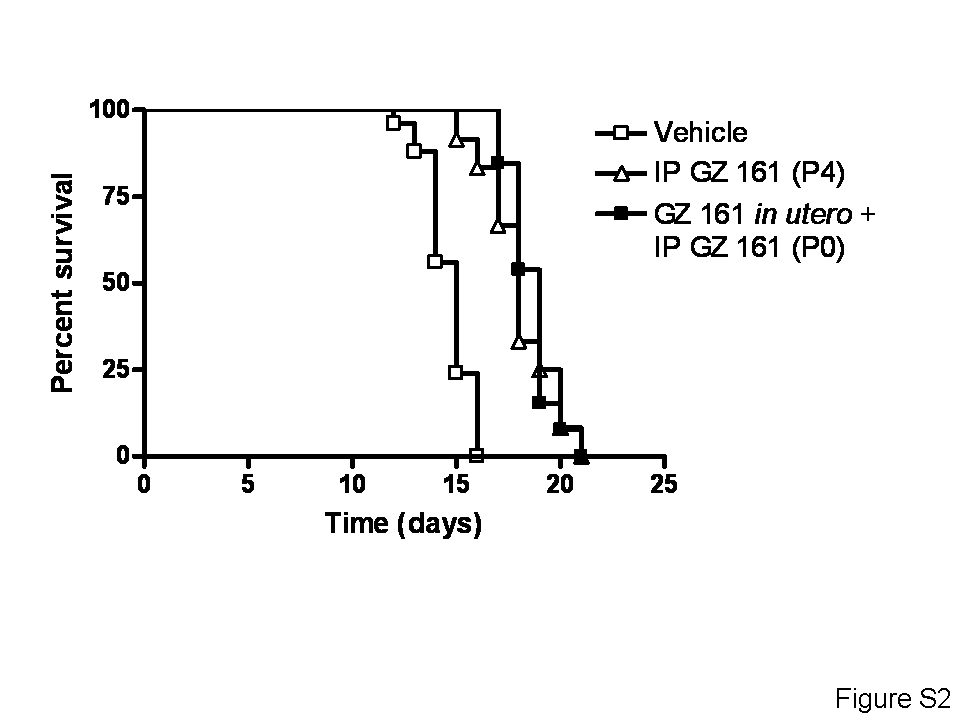

Supplement: Figure S2 — Treating K14 mice with GZ 161 in utero has a minimal effect on survival. K14 mice treated daily (IP) beginning at P4 with vehicle had a median lifespan of 14 days (N = 13). Systemic administration (20 mg/kg/day in food) of GZ 161 to pregnant heterozygote females and then daily systemic (IP) administration of GZ 161 (5 mg/kg) to the pups beginning at P0 extended lifespan to 19 days (N = 13), a result similar to treating pups daily systemically (IP) with GZ 161 at 5 mg/kg beginning at P4 (N = 12). (TIF) [file pone.0043310.s002.tif]
